# No association between markers of systemic inflammation and endothelial dysfunction with Alzheimer’s disease progression: a longitudinal study

**DOI:** 10.1007/s11357-024-01294-x

**Published:** 2024-07-31

**Authors:** Arne van Setten, Jeroen F. Uleman, René J. F. Melis, Brian Lawlor, Niels P. Riksen, Jurgen A. H. R. Claassen, Rianne A. A. de Heus, on behalf of NILVAD Study Group, Brian Lawlor, Ricardo Segurado, Sean Kennelly, Marcel G. M. Olde Rikkert, Robert Howard, Florence Pasquier, Anne Börjesson-Hanson, Magda Tsolaki, Ugo Lucca, D. William Molloy, Robert Coen, Matthias W. Riepe, János Kálmán, Rose Anne Kenny, Fiona Cregg, Sarah O’Dwyer, Cathal Walsh, Jessica Adams, Rita Banzi, Laetitia Breuilh, Leslie Daly, Suzanne Hendrix, Paul Aisen, Siobhan Gaynor, Ali Sheikhi, Diana G. Taekema, Frans R. Verhey, Raffaello Nemni, Flavio Nobili, Massimo Franceschi, Giovanni Frisoni, Orazio Zanetti, Anastasia Konsta, Orologas Anastasios, Styliani Nenopoulou, Fani Tsolaki-Tagaraki, Magdolna Pakaski, Olivier Dereeper, Vincent de la Sayette, Olivier Sénéchal, Isabelle Lavenu, Agnès Devendeville, Gauthier Calais, Fiona Crawford, Michael Mullan, Maria A. Berglund, Jurgen A. Claassen, Rianne A. de Heus, Daan L. K. de Jong, Olivier Godefroy, Siobhan Hutchinson, Aikaterini Ioannou, Michael Jonsson, Annette Kent, Jürgen Kern, Petros Nemtsas, Minoa-Kalliopi Panidou, Laila Abdullah, Daniel Paris, Angelina M. Santoso, Gerrita J. van Spijker, Martha Spiliotou, Georgia Thomoglou, Anders Wallin

**Affiliations:** 1 Department of Geriatric Medicine, Radboudumc Alzheimer Center, Radboud University Medical Center, Nijmegen, The Netherlands; 2 Copenhagen Health Complexity Center, Department of Public Health, University of Copenhagen, Oster Farimagsgade 5, 1353 Copenhagen K, Denmark; 3 Global Brain Health Institute, Trinity College, Dublin, Ireland; 4 Department of Internal Medicine, Radboud University Medical Center, Nijmegen, The Netherlands; 5 Department of Cardiovascular Sciences, University of Leicester, Leicester, UK; 6 Department of Primary and Community Care, Radboud University Medical Center, Nijmegen, The Netherlands

**Keywords:** Alzheimer’s disease, ADAS-cog, Progression, Systemic inflammation, Cytokine, Endothelial dysfunction, Biomarker, Mixed model, Longitudinal, Mediation

## Abstract

**Introduction:**

Systemic inflammation and endothelial dysfunction are potentially modifiable factors implicated in Alzheimer’s disease (AD), which offer potential therapeutic targets to slow disease progression.

**Methods:**

We investigated the relationship between baseline circulating levels of inflammatory (TNF-α, IL-1ß) and endothelial cell markers (VCAM-1, ICAM-1, E-selectin) and 18-month cognitive decline (ADAS-cog12) in 266 mild-to-moderate AD patients from the NILVAD study. We employed individual growth models to examine associations, potential mediation, and interaction effects while adjusting for confounders.

**Results:**

The average increase in ADAS-cog12 scores over all patients was 8.1 points in 18 months. No significant association was found between the markers and the rate of cognitive decline. Mediation analysis revealed no mediating role for endothelial cell markers, and interaction effects were not observed.

**Discussion:**

Our results do not support the role of systemic inflammation or endothelial dysfunction in progression in persons with AD.

**Supplementary Information:**

The online version contains supplementary material available at 10.1007/s11357-024-01294-x.

## Introduction

Alzheimer’s disease (AD) poses a tremendous burden on patients and healthcare systems and is in urgent need of safe and effective disease-modifying therapies. Therefore, it is crucial to identify potential modifiable factors that could serve as potential treatment targets to slow AD progression. Promising candidates among these factors are systemic inflammation and endothelial dysfunction [57, 63].

Several studies have suggested a potential role of systemic inflammation in AD progression. Mechanistically, systemic inflammation may affect the brain through the circumventricular organs, the blood–brain barrier, endothelial cell signaling, or the vagus nerve [2, 8, 9, 21, 22, 33, 56, 67]. By gaining access to the brain, systemic inflammatory cytokines may potentiate neuroinflammation [53, 65] and affect neuron and glial cell functioning, tau phosphorylation, amyloid beta oligomerization, and breakdown of neurotransmitters into harmful metabolites [15, 43, 45, 48, 51, 59, 65, 68], possibly leading to cognitive impairment [67]. However, although observational studies have consistently found elevated inflammatory markers in AD [58], the literature on the longitudinal impact on cognitive decline is conflicting [23, 24, 32, 40]. Consequently, the role of systemic inflammation in AD progression remains to be fully established.

Besides inflammation, vascular pathology may also play a role in AD etiology and progression [11, 30, 34, 66]. Endothelial dysfunction may contribute to cognitive impairment via impaired cerebral perfusion, increased blood–brain barrier permeability, and exposure of the brain to toxic substances, causing neuronal damage and further brain injury [27, 46, 69, 70]. Indeed, several studies found that increased levels of markers of endothelial dysfunction, such as VCAM-1 and E-selectin, are linked to worse cognitive functioning and pathophysiological markers in AD patients [25, 34, 42, 70]. However, to our knowledge, the longitudinal relationship between endothelial cell markers and cognitive decline has not yet been studied in AD patients.

In addition to their individual effects, systemic inflammation and endothelial dysfunction are exposures that are possibly associated in the etiology of cognitive decline in AD patients. Increased levels of systemic inflammatory cytokines, such as TNF-α and IL-1ß, can result in endothelial dysfunction, including blood–brain-barrier dysfunction [26, 61, 62], leading to cognitive decline. Furthermore, endothelial and blood–brain barrier dysfunction can be hypothesized to worsen the effect of systemic inflammation by increasing access to the brain [67], suggesting an interaction effect. Taken together, these observations suggest that endothelial dysfunction may mediate and moderate the effect of systemic inflammation.

In this paper, we used a prospective cohort with a follow-up period of 18 months to investigate the association between the baseline levels of the inflammatory markers TNF-α and IL-1ß and of endothelial cell markers VCAM-1, ICAM-1, and E-selectin and the rate of cognitive decline in patients with mild-to-moderate AD. Additionally, we assessed the potential mediating role of the endothelial cell markers in the hypothesized effect of the inflammatory markers and possible interaction effects between them. We hypothesized that increased baseline levels of systemic inflammation and endothelial cell markers would accelerate cognitive decline.

## Materials and methods

### Study design and participants

Data for this study were derived from the NILVAD trial [38, 39]. The NILVAD trial was a phase III randomized controlled study of the effects of nilvadipine on cognitive and functional outcomes in 511 mild-to-moderate AD patients. The results did not suggest a benefit of nilvadipine [39]. Blood samples were collected from *n* = 335 participants who consented to the ‘NILVAD blood and genetic biomarker substudy’ [49]. Data were collected by 23 academic centers in nine European countries: Ireland, the United Kingdom, Italy, the Netherlands, France, Greece, Sweden, Germany, and Hungary.

Participants were aged 50 years and older and had a diagnosis of probable AD according to the criteria of the National Institute of Neurological and Communicative Disorders and Stroke/Alzheimer’s Disease [47]. Using the National Institute on Aging- Alzheimer’s Association Research Framework, these participants would now be classified as Alzheimer’s Clinical Syndrome [29]. A complete overview of the inclusion and exclusion criteria for the NILVAD trial can be found in the study protocol [38]. Recruitment for the study was undertaken locally at each study site according to local guidelines and procedures relevant to that site [38]. The study was carried out according to the Declaration of Helsinki and approved by the appropriate National Competent Authorities, Independent Ethics Committees, and Institutional Review Boards of all participating countries.

### Blood biomarkers

This study included the following peripheral markers: IL-1ß, TNF-α, ICAM-1, VCAM-1, and E-selectin. IL-1ß and TNF-α are both key mediators for the inflammatory response. IL-1ß is a pro-inflammatory marker that is crucial for host-defense responses, while TNF-α is responsible for a diverse range of signaling events within cells leading to necrosis or apoptosis [28, 44]. The endothelial cell markers ICAM-1, VCAM-1, and E-selectin are expressed in endothelial cells in response to inflammatory response (e.g., via IL-1ß and TNF-α). These markers mediate the adhesion of leukocytes, including lymphocytes and monocytes [10, 31, 37].

### Data collection

The participants underwent cognitive assessment at baseline, three months, 12 months, and 18 months. Cognitive assessment was performed using the Alzheimer’s Disease Assessment Scale-Cognitive Subscale 12 (ADAS-cog12), the trial's primary outcome. The ADAS-cog12 ranges from 0–80 points, with higher scores indicating worse cognitive performance. [55]. Compared to the commonly used mini-mental state examination (MMSE), the ADAS-cog scale is more sensitive, reliable, and less influenced by educational level and language skills [41]. Information regarding age, sex, years of education, health status, and medication use was collected through screening of the medical history and measurements of the participant's physical health.

At baseline, 30 mL of blood was collected in *n* = 335 participants [49]. Due to limited research funds, serum levels of cytokines (IL-1ß, TNF-α, ICAM-1, VCAM-1, E-selectin) were determined in a randomly selected subset of *n* = 268 using high-sensitivity ELISA kits (Puregene® Kits; Gentra Systems, Minneapolis, Minnesota, USA) as per the manufacturer’s instructions. The plasma samples were stored at -80 degrees Celsius at local study sites until the end of the NILVAD study and stored centrally afterward. A detailed overview of sample handling can be found in the blood and genetic biomarker substudy protocol [49].

### Statistical analysis

We implemented individual growth models [60] to relate baseline levels of the inflammatory and endothelial cell markers to the rate of cognitive decline. Starting from an unconditional means model, we first explored which growth curve best fitted ADAS-cog12 trajectories. We did so by successively adding linear and quadratic growth parameters and evaluated the improvement in model fit using deviance statistics (*D*) for nested models and the Akaike information criteria (AIC) value for unnested models. We used a random intercept and random slope to capture the individual differences in ADAS-cog12 at baseline and over time. After identification of the best-fitting unconditional growth models, we added the time-invariant predictors (i.e., the markers and confounders). The markers (IL-1ß, TNF-α, ICAM-1, VCAM-1, E-selectin) were then added to the unconditional growth model to assess whether they explained the between-person variance in ADAS-cog12. These models can be used to evaluate the cross-sectional and longitudinal relationships between the markers and outcome.

To assess the potential of a mediating effect of endothelial cell markers, we employed the Baron and Kenny method [3]. This approach involved sequentially testing whether 1) inflammatory and endothelial cell marker levels individually impact cognitive progression, 2) inflammatory markers impact endothelial cell marker levels, and 3) the inclusion of the endothelial cell marker mediators alters the effects of the inflammatory markers. To assess effect modification, additional interaction terms between the markers were also added. We assessed effect modification by evaluating whether adding interaction terms between inflammatory and endothelial cell markers affected marker estimates in our models.

We adjusted for possible confounders in three steps. We first fitted models without any adjustment. We then adjusted for only age, sex, and education, and, finally, we adjusted for age, sex, education, body mass index, diabetes, vascular history, memantine intake, ACE-inhibitor intake, cholinesterase inhibitor intake (AchEI2-intake), and nilvadipine intake. Our main results are presented for the fully adjusted models. For the predictors, we standardized all numeric variables. The categorical variables, namely sex, diabetes, vascular history, memantine intake, ACE-inhibitor intake, cholinesterase inhibitor intake, and nilvadipine intake were not standardized. We also did not standardize the outcome: ADAS-cog12. We used R software version 4.0.5 with lme4 [4] to conduct the analysis.

## Results

The NILVAD trial included 511 patients, of which biomarkers were available in a subset of 268 participants. A total of 30 patients (11%) dropped out at different moments during this study and did not complete the ADAS-cog measurements: one patient for medical reasons, one patient for other reasons (unwilling, unable, agitated, measurement error), and 28 patients for unknown reasons. Out of those who dropped out, two patients did not complete any cognitive measurements at all and were, therefore, excluded from the analyses, resulting in a total of 266 patients in our analysis.

The baseline characteristics of these 266 patients are presented in Table 1. A comparison of baseline characteristics between patients who dropped out of the study and those who did not is presented in supplementary Table S1. Although patients who dropped out had higher ADAS-cog12 scores (46 points) at baseline than those who did not (33 points) (*p* < 0.0001), the inflammation and endothelial cell marker levels did not differ significantly between the groups (supplementary materials).

**Table 1:** Clinical and demographic characteristics of the study population at baseline

*Variable*	*Participants (n* = *266)*
*Female [total number, (%)]*	168 (63%)
*Age (years)*	71.8 (8.1), range: 50–87
*Education (years)*	16.4 (4.0), range: 9–29
*Body Mass Index (kg/m*^*2*^*)*	25.5 (4.3)
*Nilvadipine group [total number, (%)]*	130 (49%)
*Heart failure [total number, (%)]*	21 (8%)
*Diabetes Mellitus [total number, (%)]*	16 (6%)
*Memantine intake [total number, (%)]*	93 (35%)
*ACE-inhibitor intake [total number, (%)]*	42 (16%)
*AChEI2-inhibitor intake [total number, (%)]*	234 (87%)
*ADAS-cog12 in plaats van ADAS cog12*	
*ADAS-cog12 in plaats van ADAS cog12 score baseline (points)*	34.2 (10.4)
*ADAS-cog12 in plaats van ADAS cog12 score 3 months (points)*	35.4 (11.2)
*ADAS-cog12 in plaats van ADAS cog12 score 12 months (points)*	40.1 (13.0)
*ADAS-cog12 in plaats van ADAS cog12 score 18 months (points)*	42.3 (14.1)
*IL-1ß (pg/ml)*	0.11 (0.20)
*TNF-α (pg/ml)*	1.66 (0.66)
*ICAM-1 (pg/ml)*	339 (127)
*VCAM-1 (pg/ml)*	438 (164)
*E-selectin (pg/ml)*	7.11 (3.51)

All values are mean (standard deviation) unless otherwise specified

The average increase in ADAS-cog12 scores over all patients was 8.1 points in 18 months (i.e., 0.5 points per month; supplementary Figure S1). The range of ADAS-cog12 score changes between baseline and 18 months spanned from a reduction of 7 points (minimum) to an increase of 47 points (maximum). The best fitting model to describe the within-person changes in cognitive functioning contained a fixed and random intercept and fixed and random linear slope (*D* = 12,550.8). A model with an additional fixed and random quadratic slope was tested but did not further improve the fit of the unconditional growth model (*D* = 12,598.3). Consequently, the change in ADAS-cog12 score was modeled as linear growth with time since inclusion in the study.

The associations of inflammatory and endothelial cell marker levels with changes in ADAS-cog12 were first examined, with all markers added separately to the unconditional linear growth model (Table 2). None of the inflammatory and endothelial cell markers were significantly associated with ADAS-cog12 scores (Table 2). Without covariate adjustments, there was a significant effect of TNF-α on a decrease in ADAS-cog12, but after adjusting for age, sex, and education, this effect was no longer significant (supplementary Tables S2-S3).

**Table 2:** The results of fitting individual linear growth models with inflammatory and endothelial cell marker baseline levels on the rate of cognitive decline (ADAS-cog12)

	Unconditional		Il-1ß		TNF-α		ICAM-1		VCAM-1		E-selectin	
*Parameter*	Mean (SD)	p	Mean (SD)	p	Mean (SD)	p	Mean (SD)	p	Mean (SD)	p	Mean (SD)	p
Fixed effects												
*Intercept*^±^	34.1 (0.49)	< 0.001	30.0 (2.13)	***	29.7 (2.13)	***	30.1 (2.13)	***	30.4 (2.13)	***	30.2 (2.13)	***
Marker intercept			-0.72 (0.58)	0.22	-1.02 (0.60)	0.09	0.01 (0.60)	0.98	0.32 (0.60)	0.59	-0.56 (0.59)	0.35
*Rate of change*^*§*^	0.55 (0.02)	< 0.001	0.60 (0.12)	***	0.57 (0.12)	***	0.59 (0.12)	***	0.59 (0.12)	***	0.60 (0.12)	***
Marker rate of change			0.01 (0.03)	0.34	-0.06 (0.03)	0.05	0.01 (0.03)	0.90	0.01 (0.03)	0.68	-0.03 (0.03)	0.31
Random effects (variance components)												
Random intercept	96.1 (9.80)		77.9 (8.82)		77.5 (8.80)		78.4 (8.86)		78.3 (8.85)		78.1 (8.84)	
Rate of change	0.18 (0.43)		0.17 (0.41)		0.17 (0.41)		0.17 (0.41)		0.17 (0.41)		0.17 (0.41)	
Within person (residual)	17.7 (4.21)		16.8 (4.10)		16.8 (4.10)		16.8 (4.10)		16.8 (4.10)		16.8 (4.10)	
AIC	12,562		6940		6936		6942		6942		6941	

Except for the unconditional model, each model was adjusted for age, sex, body mass index, diabetes, education, vascular history, memantine intake, ACE-inhibitor intake, AChEI2-inhibitor intake, and nilvadipine intake

^±^For the models with IL-1ß, TNF-α, ICAM-1, VCAM-1, and E-selectin this row indicates the population average of the subjects’ individual intercepts (level-1) for participants with a time-invariant predictor value of 0 (i.e., the cross-sectional effect)

^§^For the models with IL-1ß, TNF-α, ICAM-1, VCAM-1, and E-selectin this row indicates the population average of the subjects’ individual slopes (level-1) for participants with a time-invariant predictor value of 0

^***^Indicates a *p*-value < 0.001

Although none of the markers significantly affected ADAS-cog12 scores, mediation might still occur (e.g., see [19, 20, 35, 52]). Therefore, we next performed the mediation analysis. We first assessed the effect of the inflammatory markers on the endothelial cell markers at baseline (Table 3). Both TNF-α and IL-1ß were significantly associated with VCAM-1 but not with E-selectin and ICAM-1.

**Table 3:** The results of fitting individual linear growth models with inflammatory marker baseline levels on endothelial cell marker levels

	**Independent variable**			**Dependent variable**
		Estimate	p	
IL-1ß on ICAM-1	IL-1ß	-0.05	0.38	ICAM-1
IL-1ß on VCAM-1	IL-1ß	-0.02	**0.04**	VCAM-1
IL-1ß on E-Selectin	IL-1ß	-0.04	0.54	E-selectin
TNF-α on ICAM-1	TNF-α	0.06	0.29	ICAM-1
TNF-α on VCAM-1	TNF-α	0.13	**0.04**	VCAM-1
TNF-α on E-Selectin	TNF-α	0.09	0.13	E-selectin

Each model was adjusted for age, sex, body mass index, diabetes, education, vascular history, memantine intake, ACE-inhibitor intake, AChEI2-inhibitor intake, and nilvadipine intake

We then added TNF-α or IL-1ß to the unconditional growth model with first each of the endothelial cell markers separately and then with all the markers simultaneously (Table 4). Only one of the models had a significant marker; namely, the coefficient for TNF-α was significant after adjusting for VCAM-1. However, the coefficient of TNF-α was unaltered compared to Table 2, suggesting there was no mediation by any of the endothelial cell markers.

**Table 4:** The results of fitting individual linear growth models with inflammatory and endothelial cell markers on the rate of cognitive decline (ADAS-cog12)

	**Independent variable**			**Mediator variable**					
				ICAM-1		VCAM-1		E-selectin	
		Estimate	p	Estimate	p	Estimate	p	Estimate	p
IL-1ß with mediator ICAM-1	IL-1ß	0.01	0.73	0.01	0.87				
IL-1ß with mediator VCAM-1	IL-1ß	0.01	0.69			0.02	0.64		
IL-1ß with mediator E-Selectin	IL-1ß	0.01	0.77					-0.03	0.33
IL-1ß with mediators ICAM-1, VCAM-1, AND E-Selectin	IL-1ß	0.01	0.72	0.003	0.95	0.02	0.72	-0.03	0.31
TNF-α with mediator ICAM-1	TNF-α	-0.06	0.05	0.01	0.82				
TNF-α with mediator VCAM-1	TNF-α	-0.06	**0.04**			0.02	0.51		
TNF-α with mediator E-Selectin	TNF-α	-0.06	0.06				-0.03		0.40
TNF-α with mediators ICAM-1, VCAM-1, AND E-Selectin	TNF-α	-0.06	0.05	-0.002	0.95	0.03	0.55	-0.03	0.39

Each model was adjusted for age, sex, body mass index, diabetes, education, vascular history, memantine intake, ACE-inhibitor intake, AChEI2-inhibitor intake, and nilvadipine intake. The estimate is provided for the effect of the independent variable on the dependent variable, accounting for the presence of the mediator variable

Finally, to assess potential interaction effects between inflammatory- and endothelial cell markers, we also added interaction terms between TNF-α or IL-1ß and E-selectin, ICAM-1, and VCAM-1 (supplementary Table S4). None of the interaction coefficients were statistically significant and barely affected the coefficient of TNF-α, suggesting there was no interaction between the markers either.

## Discussion

The primary objective of this study was to assess the associations between baseline levels of inflammatory and endothelial cell markers and the trajectories of cognitive decline in mild-to-moderate AD patients. Consequently, this study focused on AD progression rather than its onset. We hypothesized that patients with higher baseline marker levels would exhibit greater rates of cognitive decline. Contrary to our hypothesis, we did not find an association between levels of these markers and cognitive decline. Additionally, we found no evidence of a mediating effect of endothelial cell markers, nor any interaction effect between these markers.

Previous research yielded mixed findings regarding the effects of inflammatory and endothelial cell markers in AD. While elevated TNF-α serum levels were associated with a fourfold increase in cognitive decline in AD patients [24], three longitudinal studies did not find significant relationships between TNF-α or IL-1ß serum levels and cognitive decline [23, 32, 40]. Similarly, while some studies have identified cross-sectional relationships between plasma endothelial cell marker levels and cognitive functioning in AD patients [16, 25] and dementia-free older adults [7], these relationships were not observed longitudinally in dementia-free individuals [7]. No prior studies had investigated these relationships longitudinally in AD patients.

Our study's findings contribute to the existing literature by suggesting that inflammatory markers (TNF-α and IL-1ß) and endothelial cell markers (E-selectin, ICAM-1, and VCAM-1) have neither cross-sectional nor longitudinal associations with cognitive decline in AD patients. One possible explanation for these results is that we may not have measured the most relevant markers. IL-1ß and TNF-α are cytokines among many potential inflammatory markers, such as IL-6. Additionally, circulating cytokines do not necessarily reflect the phenotype of circulating immune cells, which may contribute to inflammation even when circulating markers appear normal [36]. Furthermore, we focused specifically on patients with mild-to-moderate AD while these markers might be most relevant in earlier disease stages, such as mild cognitive impairment or prodromal stages of AD. The follow-up duration of 1.5 years may also be seen as too short to detect an effect, even on disease progression. However, there was a clinically significant rate of disease progression in the population we studied, with an average increase in ADAS-Cog 12 of 8.1 points over 1.5 years. In context, a meta-analysis of 140 AD trials found an annual cognitive decline rate of 5.8 points on the ADAS-cog scale [71]. Despite these considerations, our findings lead us to tentatively conclude that these markers are not associated with progression. Further research is needed to explore a broader range of biomarkers, different stages of disease progression, and longer follow-up periods to fully understand the role of systemic inflammation and endothelial dysfunction in AD.

### Limitations

The present study has several limitations. Firstly, we only studied the effect of baseline marker levels on the rate of cognitive decline, while the temporal variations in cytokine levels may be substantial during disease progression [50]. Therefore, future studies could take repeated measurements of the markers to assess the potential impact of temporal variability and the timing of exposures (e.g., inflammatory markers preceding endothelial cell markers in time). Secondly, although we found comparable inflammation levels with several studies of AD patients [1, 23, 32, 64], other comparable studies found higher inflammation levels for TNF-α and IL-1ß [6, 18, 24, 40, 73]. One possible explanation for the relatively low levels in our samples could be possible protein degradation during storage of the frozen samples. In particular, IL-1ß is known to degrade during long-term storage [14]. Moreover, the quantification of IL-1ß levels in blood relies heavily on the sensitivity of the chosen assay due to its inherently low concentration in blood. Nevertheless, although studies report some differences in plasma endothelium levels in AD patients, our values are within a similar range [5, 17, 25, 54, 72], so the possible degradation is likely limited. Thirdly, although AD, MCI, and dementia diagnoses were established according to the NIA-AA guidelines using clinical, cognitive, and magnetic resonance imaging biomarkers, the diagnosis was not confirmed with amyloid biomarker evidence, This means that up to 20% of patients included in the trial may not have had significant amyloid pathology [12, 13, 39]. We also analyzed a randomized controlled trial with an observational approach. This may limit the generalizability of our results across the mild-to-moderate AD population.

## Conclusion

Contrary to our hypothesis, baseline TNF-α, IL-1ß, VCAM-1, ICAM-1, and E-selectin were not associated with accelerated cognitive decline in mild-to-moderate AD patients. The endothelial cell markers did not mediate the effect of the systemic inflammatory markers, nor was there evidence for an interaction effect. Therefore, our results do not support a role for systemic inflammation or endothelial dysfunction in AD progression.

## Supplementary Information

Below is the link to the electronic supplementary material.

Supplementary file1 (PDF 837 KB)

## Data Availability

In accordance with Irish and European data protection law, the terms under which ethical approval for the trial were granted, and the consortium agreement entered into by the NILVAD centres, we are unable to make public any patients’ personal data, even deidentified. Researchers interested in access to the trial data may contact the Trinity College Dublin Data and Material Transfer Agreements, Trinity Research and Innovation, O’Reilly Institute, Trinity College, Dublin 2, Ireland, to apply for access.
