# Supplementary material for: No association between markers of systemic inflammation and endothelial dysfunction with Alzheimer’s disease progression: a longitudinal study
Source: GeroScience. 2024 Jul 31;47(1):1093–104. doi: 10.1007/s11357-024-01294-x (PMC11872860; doi:10.1007/s11357-024-01294-x)
Supplement: Supplementary file 1 — Supplementary file1 (PDF 837 KB) [file 11357_2024_1294_MOESM1_ESM.pdf]

Supplementary materials

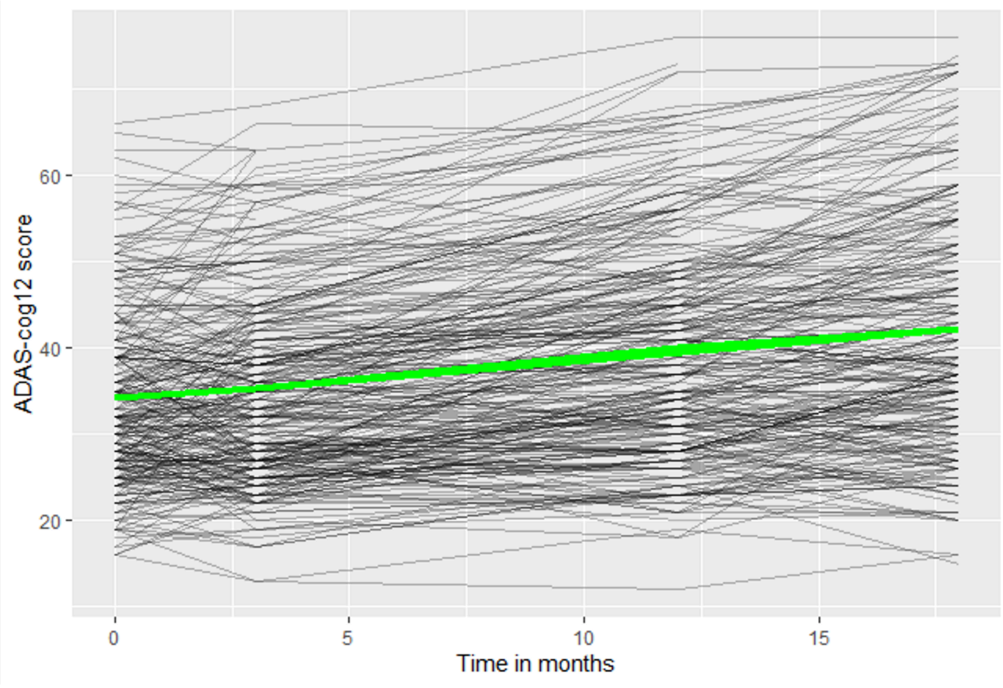

Figure S1: The cognitive decline trajectories of the mild-to-moderate AD patients included in our study (n=266). The green line represents the mean increase of ADAS-cog12 scores over all subjects

Table S1: Clinical and demographic characteristics of the study population at baseline for the total population, non-dropouts, and dropouts

Variable	***Total population (n=511)***	***Non-dropout (n=238)***	***Dropouts (n=28)***
Female [total number, (%)]	311 (61%)	146 (62%)	18 (61%)
Age (years)	72.7 (8.4), range 50-91	72.7 (8.2), range 50-87	68.5 (8.9), range: 51-82
Education (years)	16.4 (4,1), range 8-37	16.5 (4.1), range 9-29	15.8 (3.7), range: 9-24
Body Mass Index (kg/m²)	25.5 (4.2)	25.7 (4.3)	25.1 (3.7)
Heart failure [total number, (%)]	41 (8%)	17 (7%)	7 (25%)
Nilvadipine intake [total number, (%)]	250 (49%)	116 (49%)	14 (50%)
Diabetes Mellitus [total number, (%)]	40 (8%)	18 (7%)	1 (4%)
Memantine intake [total number, (%)]	130 (26%)	79 (34%)	15 (50%)
ACE-inhibitor intake [total number, (%)]	87 (17%)	35 (15%)	6 (21%)
AChEI2-inhibitor intake [total number, (%)]	450 (88%)	209 (88%)	25 (82%)
ADAS-cog12 score			
ADAS-cog12 score baseline (points)	34.5 (10.6)	32.9 (9.7)	46.1 (10.7)
ADAS-cog12 score 3 months (points)	35.3 (11.5)	33.6 (10.6)	48.3 (11.8)
ADAS-cog 12 score 12 months (points)	39.7 (13.3)	38.1 (12.4)	57 (9.50)
ADAS-cog 12 score 18 months (points)	41.8 (14.5)	41.8 (14.5)	NA
IL-1ß (pg/ml)	0.11 (0.20)	0.12 (0.22)	0.09 (0.07)
TNF-α (pg/ml)	1.66 (0.66)	1.68 (0.68)	1.54 (0.50)
ICAM-1 (pg/ml)	338 (127)	333 (122)	386 (160)
VCAM-1 (pg/ml)	437 (164)	434 (167)	475 (136)
E-selectin (pg/ml)	7.10 (3.51)	7.14 (3.38)	6.89 (4.57)

We also compared the ADAS-cog12 scores and the marker scores for the non-dropout and dropout groups using Welch's unpaired t-test. The tests for not significant for IL-1ß (p=0.13), TNF-α (p=0.19), ICAM-1 (p=0.10), VCAM-1 (p=0.15), and E-selectin (p=0.78), but highly significant for ADAS-cog12 at baseline, three months, and 12 months (p<0.0001).

Table S2: The results of fitting individual linear growth models with inflammation and endothelial cell marker baseline levels on the rate of cognitive decline (ADAS-cog12) without covariate adjustment

	**Unconditional**		**IL-1ß**		**TNF-α**		**ICAM-1**		**VCAM-1**		**E-selectin**	
**Parameter**	*Mean (SD)*	*p*	*Mean (SD)*	*p*	*Mean (SD)*	*p*	*Mean (SD)*	*p*	*Mean (SD)*	*p*	*Mean (SD)*	*p*
**Fixed effects**												
**Intercept±**	34.1 (0.49)	***	34.0 (0.62)	<0.001	34.0 (0.62)	***	34.0 (0.62)	***	34.0 (0.62)	***	33.9 (0.62)	***
**Marker intercept**			-0.62 (0.62)	0.31	-0.63 (0.62)	0.09	0.76 (0.62)	0.22	0.84 (0.62)	0.18	-0.67 (0.62)	0.28
**Rate of change§**	0.55 (0.02)	***	0.58 (0.03)	<0.001	0.57 (0.03)	***	0.58 (0.03)	***	0.58 (0.03)	***	0.58 (0.03)	***
**Marker rate of change**			0.01 (0.03)	0.90	-0.08 (0.03)	**0.02**	0.01 (0.03)	0.71	0.01 (0.03)	0.93	-0.04 (0.03)	0.20
**Random effects (variance components)**												
**Random intercept**	96.1 (9.80)		92.7 (9.63)		92.7 (9.62)		92.6 (9.62)		92.3 (9.61)		92.7 (9.62)	
**Rate of change**	0.18 (0.43)		0.20 (0.45)		0.19 (0.44)		0.20 (0.45)		0.20 (0.45)		0.20 (0.44)	)
**Within person (residual)**	17.7 (4.21)		16.9 (4.10)		16.8 (4.10)		16.8 (4.10)		16.8 (4.10)		16.8 (4.10)	
**AIC**	12562		6968		6963		6967		6967		6967	

± For the models with IL-1ß, TNF-α, ICAM-1, VCAM-1, and E-selectin this row indicates the population average of the subjects' individual intercepts (level-1) for participants with a time-invariant predictor value of 0 (i.e., the cross-sectional effect)

§ For the models with IL-1ß, TNF-α, ICAM-1, VCAM-1, and E-selectin this row indicates the population average of the subjects' individual slopes (level-1) for participants with a time-invariant predictor value of 0

**** Indicates a p-value <0.001*

Table S3: The results of fitting individual linear growth models with inflammatory and endothelial cell marker baseline levels on the rate of cognitive decline (ADAS-cog12). Except for the unconditional model, each model was adjusted for age, sex, and education

	Unconditional		***IL-1ß***		***TNF-α***		***ICAM-1***		***VCAM-1***		***E-selectin***	
Parameter	*Mean (SD)*	*p*	*Mean (SD)*	*p*	*Mean (SD)*	*p*	*Mean (SD)*	*p*	*Mean (SD)*	*p*	*Mean (SD)*	*p*
Fixed effects												
Intercept±	*34.1 (0.49)*	*****	*32.9 (1.00)*	*****	*32.9 (1.00)*	*****	*33.0 (1.00)*	*****	*33.0 (1.00)*	*****	*32.9 (1.00)*	*****
Marker intercept			*-0.54 (0.61)*	*0.38*	*-0.45 (0.62)*	*0.09*	*0.28 (0.62)*	*0.65*	*0.48 (0.62)*	*0.43*	*-0.67 (0.62)*	*0.28*
Rate of change§	*0.55 (0.02)*	*****	*0.60 (0.05)*	*****	*0.60 (0.05)*	*****	*0.60 (0.05)*	*****	*0.60 (0.05)*	*****	*0.60 (0.05)*	*****
Marker rate of change			*0.01 (0.03)*	*0.79*	*-0.05 (0.03)*	*0.10*	*0.01 (0.03)*	*0.74*	*0.02 (0.03)*	*0.58*	*-0.04 (0.03)*	*0.25*
Random effects (variance components)												
Random intercept	*96.1 (9.80)*		*87.2 (9.34)*		*87.4 (9.35)*		*87.5 (9.35)*		*87.3 (9.35)*		*87.1 (9.33)*	
Rate of change	*0.18 (0.43)*		*0.18 (0.42)*		*0.18 (0.42)*		*0.18 (0.42)*		*0.18 (0.42)*		*0.17 (0.42)*	
Within person (residual)	*17.7 (4.21)*		*16.9 (4.10)*		*16.8 (4.11)*		*16.8 (4.11)*		*16.9 (4.11)*		*16.9 (4.11)*	
AIC	*12562*		*6945*		*6943*		*6946*		*6946*		*6944*	

± For the models with IL-1ß, TNF-α, ICAM-1, VCAM-1, and E-selectin this row indicates the population average of the subjects' individual intercepts (level-1) for participants with a time-invariant predictor value of 0 (i.e., the cross-sectional effect)

§ For the models with IL-1ß, TNF-α, ICAM-1, VCAM-1, and E-selectin this row indicates the population average of the subjects' individual slopes (level-1) for participants with a time-invariant predictor value of 0

**** Indicates a p-value <0.001*

Table S4: The results of fitting individual linear growth models with inflammatory and endothelial cell marker baseline levels and an interaction term between these markers on the rate of cognitive decline (ADAS-cog12). Each model was adjusted for age, sex, body mass index, diabetes, education, vascular history, memantine intake, ACE-inhibitor intake, AChEI2-inhibitor intake, and nilvadipine intake

	Independent variable			Interaction variable					
				IL-1ß*ICAM-1		IL-1ß*VCAM-1		IL-1ß*E-selectin	
		Estimate	p	Estimate	p	Estimate	p	Estimate	p
IL-1ß with IL-1ß * ICAM-1	IL-1ß	0.06	0.19	0.15	0.15				
	ICAM-1	0.02	0.53						
IL-1ß with IL-1ß * VCAM-1	IL-1ß	0.01	0.89			-0.01	0.94		
	VCAM-1	0.01	0.69						
IL-1ß with IL-1ß * E-SELECTIN	IL-1ß	0.02	0.61					0.06	0.51
	E-selectin	-0.03	0.46						
				TNF-α*ICAM-1		TNF-α*VCAM-1		TNF-α*E-selectin	
TNF-α with TNF-α * ICAM-1	TNF-α	-0.06	0.07	0.05	0.15				
	ICAM-1	-0.01	0.85						
TNF-α with TNF-α * VCAM-1	TNF-α	-0.06	**0.04**			0.001	0.97		
	VCAM-1	0.03	0.55						
TNF-α with TNF-α * E-SELECTIN	TNF-α	-0.06	0.07					-0.02	0.49
	E-selectin	-0.03	0.40						
